# Provision of hospice and palliative care and implementation of advance care planning for residents in German nursing homes – a cross-sectional study

**DOI:** 10.1186/s12877-024-05578-x

**Published:** 2024-12-12

**Authors:** Christopher Berloge, Anna Völkel, Hannes Jacobs, Birte Burger, Jona T. Stahmeyer, Anna Levke Brütt, Falk Hoffmann, Tanja Schleef, Stephanie Stiel

**Affiliations:** 1 Institute for General Practice and Palliative Care, Hannover Medical School, Carl-Neuberg-Straße 1, Hannover, 30625 Germany; 2 Department of Health Services Research, Junior Research Group for Rehabilitation Sciences, Carl von Ossietzky Universität Oldenburg, Oldenburg, Germany; 3 Department of Health Services Research, Division of Outpatient Care and Pharmacoepidemiology, Carl von Ossietzky Universität Oldenburg, Oldenburg, Germany; 4 Health Services Research Unit, AOK Niedersachsen, Hannover, Germany; 5 Department of Medical Psychology, University Medical Center Hamburg-Eppendorf, Hamburg, Germany

**Keywords:** Advance care planning, End-of-life, Nursing home, Palliative care

## Abstract

**Background:**

Demographic changes are leading to a rise in the demand for care services, with nursing homes (NHs) playing an increasingly important role in end-of-life care. Evidence suggests that NH residents at the end of life significantly benefit from hospice and palliative care and the implementation of advance care planning (ACP). In 2018, Germany passed a law to promote the implementation of ACP in NHs and to enable the refinancing of ACP services by the statutory health insurance funds. The present study aimed at assessing current hospice and palliative care structures, examining the implementation of ACP and identifying barriers to obtain approval for billing for ACP services under this specific legislative (§ 132 g SGB V).

**Methods:**

As a part of the "Gut-Leben" project, the present study administered a cross-sectional quantitative survey in 2023 to evaluate hospice and palliative care structures, the implementation of ACP, and barriers to ACP approval in German NHs. A questionnaire was distributed to a random sample of *N* = 1,369 NH managers. Responses were analyzed using descriptive statistical methods.

**Results:**

*N* = 330 NH managers responded to the questionnaire (24.1% response). The majority described the end-of-life care structures in their region as rather good (77.7; *n* = 256), and very strong collaboration was reported with general practitioners (54.7%), physiotherapists (42.9%), and palliative care physicians (38.6%). Awareness of the legislative for ACP was reported by *n* = 201 NHs (64.4%), and 100 (50.3%) of these NHs had already obtained approval. The primary barriers to approval identified by 68 NHs included a lack of available staff (48.5%; *n* = 33) and a small facility size (27.9%; *n* = 19).

**Conclusions:**

Although half of the NHs have implemented ACP, there is still a need to enhance awareness of the ACP legislative. These efforts should aim to reduce the existing barriers to approval, which could be achieved, for instance, by fostering collaboration between NHs or by cooperating with external ACP service providers.

**Supplementary Information:**

The online version contains supplementary material available at 10.1186/s12877-024-05578-x.

## Background

Demand for residential care for the elderly is significant in industrialized nations, and demographic changes are indicating a continual rise in the number of individuals requiring long-term care [1, 2]. In Germany, as of 2021, there were 16,115 nursing homes (NHs) of various types, accommodating approximately 800,000 residents in need of care [3]. NHs are becoming increasingly important places for end-of-life (EOL) care and death. A German study by Herbst et al. (2020) revealed that the proportion of deceased individuals in NHs increased from 15.3% to 27.1% between 2007 and 2017 [4]. Further research by Walther et al. (2022) found that more than a third of NH residents died within the first year of admission [5]. Consequently, a considerable proportion of NH residents have short stays, and evidence suggests a general decrease in the length of NH stay in recent years [6].

For these reasons, the provision of hospice and palliative care for NH residents is highly relevant. Palliative care in German NHs is typically provided by general practitioners as part of general outpatient palliative care, in collaboration with in-house nursing and social services staff and with other relevant stakeholders, such as voluntary hospice services. When residents have complex palliative care needs, the involvement of specialized outpatient palliative care provided by external interdisciplinary teams is indicated [7, 8]. Admission to an inpatient hospice after NH residency is rare and considered only in exceptional cases, when the individual can no longer be adequately cared for with outpatient palliative support from GPs and specialized outpatient palliative care teams [9]. Typically, the criteria for admission to an inpatient hospice define a life-limiting disease and a prognosis of less than 6 months [10]. Consequently, NHs are designed to be the primary EOL location for most residents, while inpatient hospices are intended for situations where adequate outpatient palliative care and EOL support is unavailable in NHs or at home [11].

It is estimated that a considerable portion of dying NH residents could benefit from palliative care [12], and assumed that more frequent and robust integration of palliative care and staff training in NHs could enhance EOL care [5, 13]. Indeed, cooperation with hospice and palliative care services and providers has been shown to improve the quality of care, dying, and death in NHs. In more detail, Cimino et al. (2014) reviewed international evidence on the impact of increased understanding among practitioners of palliative and hospice care in NHs, highlighting improvements in residents' pain and symptom management, reduced hospitalization and health care costs, and higher quality care provision as perceived by family members and health care proxies [12].

A potential strategy for improving EOL besides offering hospice and palliative care in NHs globally is the implementation of advance care planning (ACP) [14]. ACP is defined as a structured and continuous process of communication between trained ACP facilitators, individuals, their next of kin and relevant healthcare providers with the aim of discussing, documenting, and, if necessary, adapting preferences for the future medical treatments and nursing care [15, 16]. This process allows for the continued consideration of a person’s healthcare preferences in the event that they are unable to express their wishes or make decisions regarding their own healthcare due to a deterioration in their health status [16]. To ensure that people who lack capacity to express or give consent receive treatment and care according to their preferences, a comprehensive institutional and regional implementation of ACP is necessary. In Germany, ACP was first tested and evaluated in a few regionally limited pilot projects in the early 2000s [17]. Based on the positive evaluation of the pilot projects, a legislative (§ 132 g Book V of the German Social Code (SGB V) – “Health Care Planning for the Last Phase of Life.”) was passed in 2015 that allows fully inpatient NHs and care facilities for individuals with disabilities to offer ACP to their residents as a service refinanced by the statutory health insurance.

Since 2018, NHs and care facilities for individuals with disabilities in Germany have been eligible for approval to provide ACP under § 132 g SGB V. To obtain approval, NHs must develop up a palliative care concept, including collaboration with both internal and external palliative care providers, and appoint a qualified ACP facilitator to conduct consultations with residents about their medical and nursing care preferences. Once approved, these consultations are subsequently reimbursed by health insurance companies, providing cost coverage equivalent to 0.25 of a facilitator’s full-time position per 100 residents [18–20]. Currently, the extent of cooperation between NHs and hospice and palliative care service providers remains unclear, and there are no available data on the implementation of this legal provision for ACP in NHs. Accordingly, the present study aimed at evaluating the current status of hospice and palliative care provision in German NHs, as well as the implementation of ACP and its barriers in this context.

## Methods

The "Gut-Leben" research project was established to investigate the current status of health care planning for the final phase of life, as stipulated by § 132 g SGB V, and to generate practical and actionable recommendations for the enhancement of EOL care within NHs [21]. The project is funded by the Innovation Fund of the Joint Federal Committee for Health Services Research (funding code 01VSF21040) for a duration of 36 months, and is divided into five work packages. The present study drew on data derived from the first work package, focused on the ACP approval processes of NHs, as well as their practical implementation of ACP and barriers to implementation.

### Study design, inclusion and exclusion criteria and sample

A cross-sectional design was employed, with a postal, standardized quantitative survey administered to NHs across Germany. Initially, data (i.e., addresses, contact details) for all 11,625 inpatient long-term care NHs in Germany were sourced from the AOK Care Navigator (“AOK Pflegenavigator”) in July 2022 [22]. Subsequently, a simple random sample of 1,400 NHs was drawn in September 2022 using IBM SPSS Statistics (Version 28). During the preparation and revision of the address lists, 31 NHs of the random sample were excluded due to incomplete contact details, duplicate addresses, pending or completed closure, or failure to meet the inclusion criterion of a fully inpatient NH (in accordance with §132 g SGB V, which authorizes the provision of ACP at the expense of statutory health insurance funds by care homes that are not day or short-term care homes). In consequence, we built a final sample of *N* = 1,369 (11.8% of the total *N* = 11,625 NHs in Germany). The sample size was selected in such a way that, after checking the plausibility of the address data, at least 10% of all long-term care facilities listed in the AOK Care Navigator could be contacted, and that the expected response rate would include at least *n* = 60 NHs that are already authorized to bill for ACP services in accordance with § 132 g SGB V.

### Development of the questionnaire and pre-testing

An interdisciplinary research team developed a four-page questionnaire for NHs (see Additional file 1), using the same structure and layout as a questionnaire for general practitioners designed in a prior study [23]. During pre-testing, the questionnaire was discussed and revised by members of the project practice advisory board (their integration in the project has been described elsewhere [21]). Subsequently, the questionnaire was tested by four NH managers using the “think aloud” technique [24].

The present study analyzed six content sections of the questionnaire, pertaining to:

1. characteristics of the responding NHs and individual respondents,
2. characteristics of the NH residents and their medical care,
3. availability of relevant EOL care structures and intensity of cooperation within the NHs,
4. availability of any ACP consultations for the NH residents (irrespective of whether the service is covered by the statutory health insurance scheme),
5. implementation of ACP services in accordance with § 132 g SGB V, and
6. barriers to obtain approval for billing ACP services under § 132 g.

The majority of questionnaire items were single-choice questions addressing aspects such as knowledge of ACP under §132 g SGB V or the organization of implementation. Additionally, some items solicited estimates of percentages regarding the prevalence of oncological diseases among residents or the presence of particular individuals during ACP consultations (such as relatives, legal guardians, attending physicians, the resident concerned and/or NH staff). Section 3 queried the availability of relevant EOL (i.e., palliative and hospice) care structures in the local region and asked respondents to rate the intensity of cooperation with these structures on a 5-point Likert scale ranging from 0 (none) to 4 (very strong). The section concluded with a question regarding the overall assessment of the palliative care structures in the region. Four potential responses (very poor – rather poor – rather good – very good) were provided. The objective of section 6 was to identify the barriers to obtain approval for billing ACP services at the expense of the statutory health insurance funds. The initial section was directed to NHs that had already been approved under § 132 g and sought to identify the primary obstacles encountered in the process of obtaining approval through a free text field. The subsequent part addressed NHs that had elected not to pursue approval for billing ACP services, inquiring about the rationale behind this decision. To facilitate comprehensive responses, a range of potential answers was provided, as well as an additional free text field.

### Survey implementation

Initially, 1,369 questionnaires were sent with personalized cover letters to the relevant NH management staff, alongside an information sheet about the project. The package included a self-addressed stamped envelope for the return of the questionnaire, which did not require any address details or designation of the participating NH. A reminder was sent 3 weeks later (February 2023). The entire process, from the first mailing to the receipt of the final response (June 2023), spanned 17 weeks.

### Data analysis

The anonymized data from the returned questionnaires were entered into an IBM SPSS Statistics (Version 28) database, and a double data check was performed to eliminate false entries. The data analysis incorporated descriptive and frequency statistics. The results are reported here as means with standard deviation (SD), ranges (minimum–maximum), or absolute (n) and relative frequencies (%). Free text responses were also considered in the interpretation of the results.

The present study received a waiver from the local medical ethics committee of the Carl von Ossietzky Universität of Oldenburg (no. 2022–154). Since data was collected anonymously, consent to participate was not required.

## Results

### Characteristics of responding NHs

Out of the 1,369 NHs contacted, 330 returned the questionnaires, corresponding to a response of 24.1%. The average age of the respondents was 49.7 years, and the majority were female (70.9%, 229/323). Additionally, 59.7% (194/325) were facility managers (see Table 1).

**Table 1:** Characteristics of the responding NHs (*N* = 330)

**Respondent characteristics**		
**Respondent age in years (*n* = 316)**^**a**^ mean (SD)	49.7 (10.2)	
	%	*n*
≤ 40	22.2	70
41–60	66.1	209
> 60	11.7	37
**Respondent sex (*n* = 323)**^**a**^	%	*n*
Male	28.2	91
Female	70.9	229
Diverse	0.9	3
**Respondent position in NH (*n* = 325)**^**a**^ (multiple answers allowed)	%	*n*
Nursing staff management	28.3	92
Facility management	59.7	194
Business management	8.8	29
Other	13.3	44
**Years in current position (*n* = 320)**^**a**^ mean (SD)	10.3 (8.3)	
**NH characteristics**		
**Type of sponsorship (*n* = 322)**^**a**^	%	*n*
Private	36.0	116
Non-profit	57.1	184
Municipal	6.8	22
**Number of beds (*n* = 322)**^**a**^	%	*n*
≤ 50	21.1	68
51–100	50.9	164
101–150	21.1	68
151–200	5.0	16
> 200	1.9	6
**Location (*n* = 318)**^**a**^	%	*n*
Rural ≤ 5,000	19.2	61
Small town > 5,000–20,000	38.7	123
Semi-urban > 20,000–100,000	20.4	65
Urban > 100,000	21.7	69
**Distance to the nearest hospital with an emergency department (*n* = 265)**^**a**^** in kilometers, mean (SD)**	8.8 (7.3)	
	%	*n*
≤ 1	15.1	40
> 1–5	32.1	85
> 5–10	18.9	50
> 10–20	29.4	78
> 20	4.5	12

^a^Numbers differ due to missing values; *SD* Standard deviation, *NH* Nursing home

The responding NHs were categorized into private (36.0%; 116/322), non-profit (57.1%; 184/322), and municipal (6.8%; 22/322) institutions. NH size was determined by the number of beds, averaging 85 per facility (range: 14–298 beds, SD: 42.6). Of these, an average of 4.4 beds (range: 0–27 beds) were allocated for short-term care. The average distance from NHs to the nearest hospital with an emergency department was 8.8 km (range: 0–35 km) (see Table 1).

### Characteristics of NH residents and their medical care

The average age of residents upon NH admission was 81.1 years (range: 50–90 years). The estimated proportion of residents with statutory health insurance was 88.4% (range: 0–100%), and those diagnosed with dementia constituted 55.4% (range: 0–100%). Furthermore, 38.4% of residents (range: 1–100%) were at care levels 4 or 5, representing the highest levels of care in Germany. Over the prior year, 38.1% of residents (range: 0–100%) had been hospitalized as an inpatient, 10.9% (range: 0–80%) had an oncological disease, and 7.7% (range: 0–85%) were bedridden. On average, each NH had 7.9 general practitioners (GPs) providing care for the residents (range: 1–40). Residents had an estimated mean 21.6 GP contacts annually (range: 1–78) (see Table 2).

**Table 2:** Characteristics of residents and their medical care (*N* = 330)

**Characteristics of residents**		
**Age at NH admission (*n* = 288)**^**a**^ mean (SD)	81.1 (6.6)	
	%	*n*
≤ 69	5.9	17
70–79	14.6	42
≥ 80	79.5	229
**Percentage with…**	mean % (SD)	
Oncological disease (*n* = 300)^a^	10.9 (11.1)	
Dementia (*n* = 322)^a^	55.4 (22.9)	
Care level 4 or 5 (*n* = 318)^a^	38.4 (20.5)	
Bedridden (*n* = 303)^a^	7.7 (11.1)	
Statutory health insurance (*n* = 316)^a^	88.4 (16.7)	
Inpatient hospitalization in the past year (*n* = 290)^a^	38.1 (24.4)	
**GP care**		
**Number of GPs per NH (*n* = 321)**^**a**^ mean (SD)	7.9 (6.8)	
	%	*n*
1–5	45.5	146
6–10	35.2	113
11–15	9.3	30
> 15	10.0	32
**Resident contact with GPs, annually (*n* = 310)**^**a**^ mean (SD)	21.6 (16.1)	

^a^Numbers differ due to missing values; *SD* Standard deviation, *NH* Nursing home, *GP* General practitioner

### Availability of EOL care structures and intensity of cooperation

The EOL care structures in the respective regions were rated as rather good by 62.1% (198/319) and very good by 18.2% (58/319) of the responding NHs. Conversely, 19.4% (62/319) rated them as rather poor and 0.3% (1/319) as very poor.

**Fig. 1:**
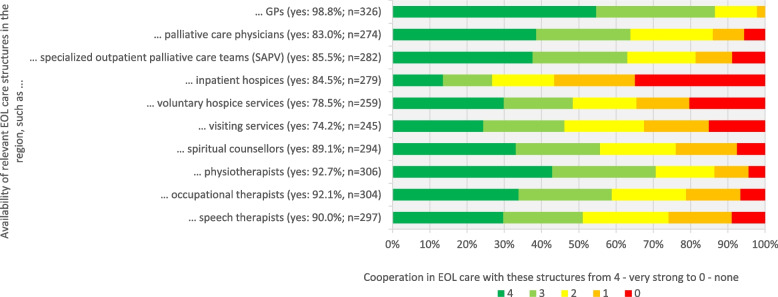
Availability of relevant EOL care structures and intensity of cooperation. GP: general practitioner; EOL: end-of-life

In terms of service availability, the majority of respondents confirmed the availability of GPs (98.8%), physiotherapists (92.7%), occupational therapists (92.1%), and speech therapists (90.0%). Lower availability was reported for visiting services (74.2%), voluntary hospice services (78.5%), and palliative care physicians (83.0%). Regarding the intensity of cooperation, very strong cooperation was reported with GPs (54.7%), physiotherapists (42.9%), and palliative care physicians (38.6%). However, NHs reported no cooperation with inpatient hospices (34.9%), voluntary hospice services (20.3%), and visiting services (15.1%) (see Fig. 1).

### ACP for German NH residents

Just over half of the surveyed facilities (52.4%, 167/319) reported some form of ACP consultation. On average, at least one ACP consultation was reported for an estimated average of 42.0% of the residents. According to the NH respondents, residents were present in 60.3% of these consultations, relatives and dependents in 58.5%, other employees in 45.6%, attending physicians in 41.5%, and legal guardians in 36.5% (see Table 3).

**Table 3:** ACP consultation provision and characteristics

Any ACP consultation in NH (*N* = 319)		
	%	*n*
Yes	52.4	167
No	47.6	152
**Estimated percentage of residents receiving ACP consultation**		
	mean % (SD)	
at least one (*n* = 159)^a^	42.0 (33.7)	
**Estimated percentage of consultations in which the following persons are in attendance**		
	mean % (SD)	
Relatives and dependents (*n* = 157)^a^	58.5 (36.2)	
Legal guardians (*n* = 138)^a^	36.5 (37.9)	
Attending physicians (*n* = 144)^a^	41.5 (39.2)	
Involved residents (*n* = 153)^a^	60.3 (38.4)	
Further employees (*n* = 117)^a^	45.6 (40.1)	

^a^Numbers differ due to missing values; *SD* Standard deviation, *ACP* Advance care planning

### Implementation of ACP under § 132 g SGB V for German NH residents

Among the responding NHs, 35.6% (111/312) were unaware of the new legislative on options for ACP. Of the 219 NHs aware of the legislative for billing for ACP services, 50.3% (100/199) reported that they are approved to bill for ACP services in accordance with the requisite approval conditions. Conversely, 49.7% (99/199) did not hold the status to bill for ACP services (see Table 4).

**Table 4:** ACP provision and characteristics (*N* = 330)

**Awareness of ACP provision under § 132 g Book V of the German Social Code (SGB V) – “Health Care Planning for the Last Phase of Life” (*n* = 312)**^a^		
	%	*n*
Yes	64.4	201
No	35.6	111
**NH approved under § 132 g (*n* = 199)**^a^		
	%	*n*
Yes	50.3	100
No	49.7	99
**Length of time for which NH has been approved under § 132 g (*n* = 65)**^a^		
	%	*n*
< 1 year	33.8	22
1–3 years	29.2	19
> 3 years	36.9	24
**Responsibility for the organization of ACP consultations under § 132 g (*n* = 96)**^a^		
	%	*n*
Qualified staff of the NH	60.4	58
Qualified staff of the NH sponsor	24.0	23
Qualified staff of an external provider	7.3	7
Qualified staff of the NH and an external provider	7.3	7
Qualified staff of the NH, the NH sponsor, and an external provider	1.0	1
**Number of facilitators offering ACP services (*n* = 96)**^a^		
	%	*n*
One	72.9	70
Two	18.8	18
Three	5.2	5
Four or more	3.1	3
**Occurrence of ACP case conferences (*n* = 93)**^a^		
	%	*n*
Yes	91.4	85
No	8.6	8

^a^Numbers differ due to missing values; *ACP* Advance care planning, *NH* Nursing home; rounding differences to 100% possible

In the majority of the 100 approved NHs, ACP was conducted by qualified staff within the facility (60.4%; 58/96), while 24.0% (23/96) relied on qualified staff from their sponsor. In 72.9% (70/96) of the NHs, a single facilitator conducted ACP consultations, whereas 27.1% (26/96) had two or more facilitators. Case conferences were included in ACP in 91.4% (85/93) of the NHs (see Table 4).

Thirty-one free-text responses referenced barriers that emerged during the approval process. These generally pertained to two thematic areas: (i) “approval requirements” (12 barriers), which cited a lack of suitable and available staff for ACP consultations, the time required to qualify staff, coordination problems with staff training centers and the development of a palliative care concept; and (ii) “approval procedures” (15 barriers), which referenced difficult cooperation with health insurance companies, the duration of the approval procedure and excessive bureaucracy.

### Barriers as reasons for not applying for approval

**Fig. 2:**
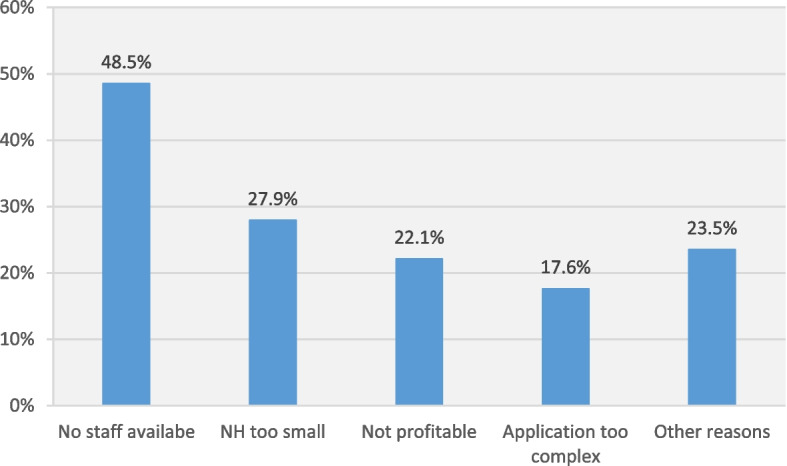
Barriers to approval under § 132 g SGB V (*N* = 68). Multiple answers possible; NH: nursing home

Among the NHs that were not approved for ACP under § 132 g SGB V, 73.9% (68/92) indicated no plans to seek future approval, while 23.9% (22/92) were planning to apply and 2.2% (2/92) had initiated the approval process. The primary reason reported for a disinclination to apply for approval was a lack of available staff (48.5%; 33/68). Additionally, 27.9% (19/68) cited the small size of their facility, 22.1% (15/68) stated it would not be profitable, 17.6% (12/68) found the application process too complex, and 23.5% (16/68) mentioned other reasons (see Fig. 2).

In 16 free-text responses, NH respondents indicated that existing structures were sufficiently robust for ACP or that alternative solutions were already being planned, making the formal implementation of ACP according to § 132 g SGB V unnecessary.

## Discussion

As part of the "Gut-Leben" project, the present cross-sectional study aimed at investigating the current status of hospice and palliative care provision and the implementation of ACP for residents in German NHs. Additionally, the study sought to generate insights into the barriers that prevent NHs from applying for statutory approval to offer ACP.

### Availability of relevant EOL care structures and intensity of cooperation

A significant finding was the intensive collaboration between almost all NHs and GPs. This is consistent with the nature of outpatient primary medical care provided by GPs in NHs and the regular contact between almost all residents and their GPs, as described in several studies [25–27]. Close interprofessional collaboration is needed to guarantee high-quality care for residents in NHs [28, 29]. This is especially crucial in the context of palliative care, which often requires a multidisciplinary approach to ensure optimal care at the end of life [7, 30]. A national survey conducted in Germany in 2018 revealed that, although some NHs sought external expertise in EOL care, collaboration and networking with other palliative care providers remains limited [7]. Just over half of the NHs surveyed reported being members of a hospice and palliative care network, with outpatient (voluntary) hospice services representing the most common cooperation partner [7]. A recent study by Walther et al. (2022) aimed at assessing the current status of palliative care in NHs of a non-profit provider in Lower Saxony, Germany, painted a rather positive picture of palliative care. A total of 14% of deceased residents were accompanied by a volunteer hospice service, while 16% of residents with an oncological disease were supported by a specialized palliative home care service [5]. However, despite the growing prevalence of palliative care concepts in NHs, there appears to be considerable variation in the availability and quality of palliative care and hospice support across the NHs [5, 7, 31]. In the present study, numerous palliative and hospice care structures for EOL care were available to NHs. However, cooperation with inpatient hospices was notably less intense. This imbalance may have stemmed from the specifics of the German health care system, wherein NH beds are generally intended for use until the resident’s EOL, making transfers to inpatient hospices less common and extensive cooperation between NHs and hospices unnecessary.

### ACP provision and implementation under § 132 g SGB V for German NH residents

Nearly a third of the surveyed NHs were unaware of the legal provisions of § 132 g SGB V for ACP in NHs. Our data did not elucidate the reasons for this. Nonetheless, despite the low level of awareness, 50% of the surveyed NHs offered some form of ACP to their residents. Considering that NHs are facilities for EOL care, in which residents' care preferences should be treated as paramount, this percentage appears relatively low. However, our findings are consistent with those of Walther et al. (2022), who reported that only 46% of NH residents in their German sample were offered ACP consultations [5]. It is conceivable that a significant proportion of NH residents may not engage in ACP due to their overall declining health, the presence of dementia, staff shortages, a lack of staff training, or personal preference [32]. Nevertheless, a study from the United Kingdom has indicated a considerably higher percentage of NH residents opting for ACP, up to 79.5% [33]. Further information campaigns about ACP under § 132 g SGB V may encourage more NHs in Germany to implement this service.

It is noteworthy that, in the case of ACP consultations, only three out of five residents were involved, as evidenced by the results of our survey. This may be attributable to the high prevalence of dementia among NH residents (i.e., more than half of the present sample), with many represented by relatives or legal guardians. According to a systematic review by Seitz et al. (2010), the median prevalence of dementia in long-term care homes in 2010 was 58% [34]. However, a recent systematic review indicates that this rate may currently be as high as 85% [35]. Evidence from a Belgian study suggests that residents with dementia wish to participate in ACP consultations themselves [36], and that participation in ACP is associated with increased satisfaction with care from the perspective of both nursing staff and the individuals concerned [37]. In light of the high prevalence of dementia, there appears to be scope for improvement in enabling residents with dementia to participate personally by adapting the style and content of ACP conversations to align with their cognitive abilities [38]. However, given that NH residents frequently present with severe forms of dementia accompanied by other non-cognitive symptoms [39], which impede their ability to engage in the process, this is more likely to apply to mild forms of dementia.

### Barriers to approval

Of note, a third of the responding NHs were not approved for the provision of ACP under § 132 g SGB V, despite being aware of these legal provisions. We assume that some NHs deemed the implementation and refinancing of ACP according to this regulation unnecessary because they were already offering ACP to their residents. The primary reasons cited for a disinclination to seek approval were insufficient staffing and the small size of the facility. The literature reports similar barriers to ACP provision, such as a lack of staff education and insufficient time [40]. To address these challenges, non-approved NHs could consider sharing staff with other NHs or partnering with external providers able to conduct in-house consultations. However, the implementation of ACP in NHs has so far been characterized primarily by the approach of training selected staff in the facilities to become ACP facilitators [41]. In response to the aforementioned challenges, In der Schmitten et al. (2022) propose inter-institutional cooperation and the deployment of a regionally based team of ACP facilitators as a potential solution. This could serve to counter the problem that the costly qualification in small facilities is considered uneconomical due to the small, part-time position that is refinanced. Furthermore, staff turnover or extended periods of absences due to illness can be more effectively managed without the ACP offering and its implementation in the NH being jeopardized by its concentration on a specific person [41].

In particular, these approaches could optimize staff resources and enhance access to ACP for NH residents. In addition, reduced bureaucratic hurdles in the application process could incentivize more NHs to seek approval.

### Strengths and limitations

We received questionnaires from all German federal states and from NHs of very different sizes. The average size of the participating NHs (based on the number of beds), the distribution of the different types of sponsorship and the average distance to the nearest hospital are in line with the figures reported in other German studies [5, 7, 42, 43], Therefore, we conclude that the respondents might represented a fairly representative cross-section of NHs in Germany. However, the response was slightly lower than that observed in comparable NH surveys [42, 43].

It is important to acknowledge the overrepresentation of responses from NHs approved under § 132 g SGB V to bill for ACP services in the present sample compared to the national percentage of approved NHs, which may reflect a positive selection bias. In Germany, only 15.3% (1,773) of all NHs are approved to bill for ACP at the expense of the statutory health insurance funds [44], whereas 30.3% of the NHs in our sample had this approval. It is plausible that NHs that were more proactive in providing of palliative care and ACP services were more likely to participate in the survey [4]. Nevertheless, the high number of NHs approved according to § 132 g SGB V in our sample allowed us to gain a more comprehensive understanding of the implementation of the legal framework for ACP services in NHs, which facilitates more precise conclusions regarding ACP in NH residents.

Finally, some of the present findings, particularly those pertaining to the estimated proportions of residents receiving ACP consultations and the estimates on individuals present during an ACP consultation, should be interpreted with caution. These data are based on the personal impressions of respondents and may not be entirely accurate. However, as the data was collected anonymously, it may be assumed that the respondents answered the questions to the best of their knowledge and that their answers may not have been biased in order to present one´s NH more positively. Furthermore, the estimated proportions of residents who have received ACP consultations are in close alignment with the data from Walther et al. regarding the offer of ACP consultations [5]. In the absence of data pertaining to the proportion of individuals involved in ACP consultations it is not possible to ascertain the reliability of these estimates.

## Conclusions

The present study aimed to assess the current status of hospice and palliative care provision in German NHs and was the first to analyze the implementation of ACP and the barriers to its implementation in this context. Overall, approximately one-third of the responding NHs demonstrated a lack of familiarity with the legislative of § 132 g SGB V for getting ACP refinanced by health insurances. Regardless of their level of awareness, only half of the NHs offered their residents some form of ACP. The findings of our study indicate that ongoing improvements in the implementation of ACP are still necessary. This concerns, on the one hand, awareness of ACP and the associated billing options, as well as the primary barriers to NH approval which could be mitigated through partnerships among NHs or between NHs and external providers of ACP consultations. Conversely, targeted initiatives may be required to facilitate greater resident involvement in ACP consultations pertaining to EOL care preferences.

## Supplementary Information

Supplementary Material 1.

## Data Availability

The datasets used and/or analyzed during the current study are available from the corresponding author upon reasonable request.

## Abbreviations

NH: Nursing homes
EOL: End-of-life
ACP: Advance care planning
SGB V: German Social Code, Book V
SD: Standard deviation
GP: General practitioner
