# Supplementary material for: Provision of hospice and palliative care and implementation of advance care planning for residents in German nursing homes – a cross-sectional study
Source: BMC Geriatr. 2024 Dec 12;24:999. doi: 10.1186/s12877-024-05578-x (PMC11636033; doi:10.1186/s12877-024-05578-x)
Supplement: Supplementary file 1 — Supplementary Material 1. [file 12877_2024_5578_MOESM1_ESM.pdf]

# Care in the last phase of life in nursing homes

**If you have any questions, please contact:**

Carl von Ossietzky Universität Oldenburg

Medizinische Hochschule Hannover

Tanja Schleef | Hannover Medical School
Institute for General Practice and Palliative Care
Carl-Neuberg-Str 1, 30625 Hannover
Tel.: 0511 xxx xxxx | E-Mail: Schleef.Tanja@mh-hannover.de

This questionnaire is intended for the management of your nursing home, e.g. the facility management, the nursing staff management or the managing director, and applies to your entire facility.

## Care in the last phase of life

**1** **Do you agree with the statement that the residents of your facility require a high level of care (e.g. medical, nursing, psychosocial) in the last phase of life?**

☐ yes ☐ no

**2** **What structures relevant to end-of-life care are available in your region and how do you assess the intensity of cooperation in care?**
(Please tick all that apply.)

| | Availability | | | Cooperation | | | | |
|---|---|---|---|---|---|---|---|---|
| | no | yes | | none 0 | 1 | 2 | 3 | very strong 4 |
| General practitioners | ☐ | ☐ | → | ☐ | ☐ | ☐ | ☐ | ☐ |
| Palliativ care physicians | ☐ | ☐ | → | ☐ | ☐ | ☐ | ☐ | ☐ |
| Specialized palliative care teams | ☐ | ☐ | → | ☐ | ☐ | ☐ | ☐ | ☐ |
| Inpatient hospices | ☐ | ☐ | → | ☐ | ☐ | ☐ | ☐ | ☐ |
| Voluntary hospice services | ☐ | ☐ | → | ☐ | ☐ | ☐ | ☐ | ☐ |
| Visiting services | ☐ | ☐ | → | ☐ | ☐ | ☐ | ☐ | ☐ |
| Spiritual counsellors | ☐ | ☐ | → | ☐ | ☐ | ☐ | ☐ | ☐ |
| Physiotherapists | ☐ | ☐ | → | ☐ | ☐ | ☐ | ☐ | ☐ |
| Occupational therapists | ☐ | ☐ | → | ☐ | ☐ | ☐ | ☐ | ☐ |
| Speech therapists | ☐ | ☐ | → | ☐ | ☐ | ☐ | ☐ | ☐ |

**How would you evaluate the structures for care in the last phase of life in your region as a whole?**

☐ very poor ☐ rather poor ☐ rather good ☐ very good

**3** **Does your facility offer any ACP services (e.g. GVP, BVP, etc.)?**

☐ yes ☐ no

→ **If yes: What proportion of residents in your facility have had at least one ACP consultation?** ………… % of residents

**Please estimate for your facility: What proportion of ACP consultations involve the following people?**

Relatives and dependents: ………… %
Legal guardians: ………… %
Attending physicians: ………… %
Residents concerned: ………… %
Further employees: ………… %

4 **Are you aware of the ACP provision under § 132g SGB V?**

yes ☐ no ☐ → *continue with question 6*

5 **Is your facility approved under § 132g SGB V?**

☐ yes → **If yes, when was the approval granted?** since: …………/………… (month/year)

**How is the provision of ACP consultations for the last phase of life organized in your facility?**

- ☐ Qualified staff from your own facility conduct ACP consultations
- ☐ Qualified staff from your facility sponsor conduct ACP consultations
- ☐ ACP consultations are conducted in collaboration with external providers

**How many facilitators provide ACP services in your facility?** Number: ………… Facilitators

→ **How many of the facilitators providing ACP in your facility have completed the 1st <u>and</u> 2nd part of the required further training?**
Number: ………… Facilitators

**Do case conferences take place in your facility as part of ACP services?**

☐ yes ☐ no

**What was the biggest barrier to approval?** ……………………………………………………

……………………………………………………………………………………………………

……………………………………………………………………………………………………

**In your opinion: Has ACP improved the care of the residents in your facility?**

☐ rather yes ☐ rather no

☐ no → **If no, what applies to your facility?**

☐ The approval is <u>not planned</u> because... (Please tick all that apply.)

- ☐ Our facility is too small
- ☐ Lack of available staff
- ☐ The billing of the service is not profitable
- ☐ The approval process is too complex
- ☐ Other reasons: ……………………………………………………………………………
……………………………………………………………………………………………………
……………………………………………………………………………………………………

☐ The approval is <u>planned</u>.

☐ The approval <u>has been pending</u> since …………/………… (month/year).
The future provision of ACP consultations will be organized as follows:

- ☐ Qualified staff from your own facility conduct ACP consultations
- ☐ Qualified staff from your facility sponsor conduct ACP consultations
- ☐ ACP consultations are conducted in collaboration with external providers

## Characteristics of NH residents and their medical care

6

**Average age of residents upon admission to the nursing home:** ……… years (based on the year 2022)

**Please estimate the percentage of residents in your facility...**

with an oncological disease: …………%

with dementia: ………… %

with care level 4 or 5: ………… %

who are bedridden: ………… %

with statutory health insurance: ………… %

who have had at least one inpatient hospitalization in the past year: ………… %

**Please estimate the proportion of female residents in your facility with…**

transurethral (via the urethra) indwelling urinary catheter: ………… %

suprapubic (puncture of the urinary bladder above the pubic bone) indwelling catheter: ………… %

7

**The following questions pertain exclusively to male residents of your facility and concern the use of transurethral and suprapubic indwelling catheters:**

| | Transurethral indwelling catheter | Suprapubic indwelling catheter |
|---|---|---|
| **What proportion** of men have a catheter? | ………… % of men | ………… % of men |
| **Which health care professional is most likely to** change catheters in men? | ☐ Nursing staff of the NH<br>☐ General practitioner<br>☐ Urologist<br>☐ Other, namely:<br>………………………………… | ☐ Nursing staff of the NH<br>☐ General practitioner<br>☐ Urologist<br>☐ Other, namely:<br>……………………………… |
| **What proportion** of men with catheters have their catheter changed **in the nursing home?** | For ………… % of men with catheters | For ………… % of men with catheters |

8

**How many general practitioners provide care for the residents of your facility?** Number: …………

**How many GP contacts does each individual resident have on average per year (both personal contacts and (video) telephone contacts)?** approx. ………… contacts per resident per year

9

**What proportion of residents in your facility have the following documents?**

Guardianship directive: ………… %

Health care proxy: ………… %

Advance directive: ………… %

Plan for emergency situations: ………… %

General power of attorney: ………… %

**How often are these documents meaningful with regard to…**

preferences in the event of hospital transfers in the last phase of life? ………… %

preferences in the event of cardiac arrest? ………… %

## Details of your facility

10

**Sponsorship**:

☐ Non-profit, church ☐ Non-profit, other ☐ Private ☐ Municipal

**Total number of beds:** ………… beds

↳ **Of these, beds for short-term care:** ………… beds

**Does your facility have at least one psychogeriatric living area (for dementia care, etc.)?**

☐ yes ☐ no

**In which federal state is your facility located?** ……………………………………………………………

**What is the size of the city where your facility is located?**

☐ Up to 5,000 inhabitants ☐ Over 5,000 to 20,000 inhabitants

☐ Over 20,000 to 100,000 inhabitants ☐ Over 100,000 inhabitants

**How far away is he nearest hospital with an emergency department from your facility?** approx. ………… km

11

**How many people are currently employed in your facility in the respective areas?**

Care: ………… Housekeeping: ………… Social services: ………… Other: …………

**How many vacant full-time or permanent positions are there currently in your facility?**

Care: ………… Housekeeping: ………… Social services: ………… Other: …………

12

**Does the management team of your facility have the following additional qualifications in the field of hospice culture and palliative competence?**

| | Further training Palliative Care (160 hours) | Basic course Palliative Care (40 hours) | Individual continuing education courses on palliative/hospice topics | none |
|---|---|---|---|---|
| Nursing staff manager | ☐ | ☐ | ☐ | ☐ |
| Social services manager | ☐ | ☐ | ☐ | ☐ |

13

**How many employees or volunteers in your facility have additional qualifications in the field of hospice culture and palliative competence?**

| | Further training Palliative Care (160 hours) | Basic course Palliative Care (40 hours) | Individual continuing education courses on palliative/hospice topics |
|---|---|---|---|
| Nursing staff | | | |
| Social services | | | |
| Visiting services | | | |

## Personal details

14

**Age**: ………… years

**Gender**: ☐ male ☐ female ☐ non-binary

**Your current position:**

☐ Nursing staff management ☐ Nursing home management ☐ Managing director

☐ Other, namely: ………………………………………………

**How long have you held this position?** ………… years

**Thank you for your participation!**
